# A Fatal Case of Primary Amoebic Meningoencephalitis from Recreational Waters

**DOI:** 10.1155/2020/9235794

**Published:** 2020-05-28

**Authors:** Edward Hamaty, Saif Faiek, Minesh Nandi, David Stidd, Manish Trivedi, Hari Kandukuri

**Affiliations:** ^1^Department of Medicine, AtlantiCare Regional Medical Center, Atlantic City, NJ, USA; ^2^Department of Critical Care, AtlantiCare Regional Medical Center, Atlantic City, NJ, USA; ^3^Division of Neuroscience, AtlantiCare Regional Medical Center, Atlantic City, NJ, USA; ^4^Department of Neurological Surgery, Thomas Jefferson University and Jefferson Hospital for Neuroscience, Philadelphia, PA, USA; ^5^Division of Infectious Disease, AtlantiCare Regional Medical Center, Atlantic City, NJ, USA

## Abstract

**Background:**

Naegleria Fowleri is a single-cell, thermophilic amphizoid amoeba, and a rare known causative agent for primary amoebic meningoencephalitis with >97% mortality rate. The amoeba resides in freshwater lakes and ponds but can also survive in inadequately chlorinated pools and recreational waters. The mode of infection includes activities such as diving or jumping into freshwater or submerging the head under the water. Although most commonly seen in the southern United States, it is essential to keep this clinical suspicion in mind regardless of geography, as presenting symptoms can be very similar to classic bacterial meningitis. *Case Summary*. We report the first-ever case in the state of New Jersey of a 29-year-old male presented after a visit to a recreational water park in Texas five days before his presentation with altered mental status. In ICU, his ICP remained refractory to multiple therapies, including antibiotics and antivirals, external ventriculostomy drain, hypertonic saline, pentobarbital-induced coma, and bilateral hemicraniectomies. The CSF analysis revealed trophozoites indicating a protozoan infection, which we diagnosed in the neurocritical unit, and the patient was then immediately started with treatment that included amphotericin B, rifampin, azithromycin, and fluconazole. This suspicion was promptly confirmed by the Center for Disease Control (CDC). Unfortunately, despite all the aggressive intervention by the multidisciplinary team, the patient did not survive.

**Conclusion:**

As per the CDC, only four people out of 143 known infected individuals in the United States from 1962 to 2017 have survived. Symptoms start with a median of 5 days after exposure to contaminated water. Given the rarity of this case and its very high mortality rate, it is crucial to diagnose primary amoebic meningoencephalitis accurately as its presentation can mimic bacterial meningitis. It is vital to obtain a careful and thorough history, as it can aid in prompt diagnosis and treatment.

## 1. Introduction

**Figure 1:**
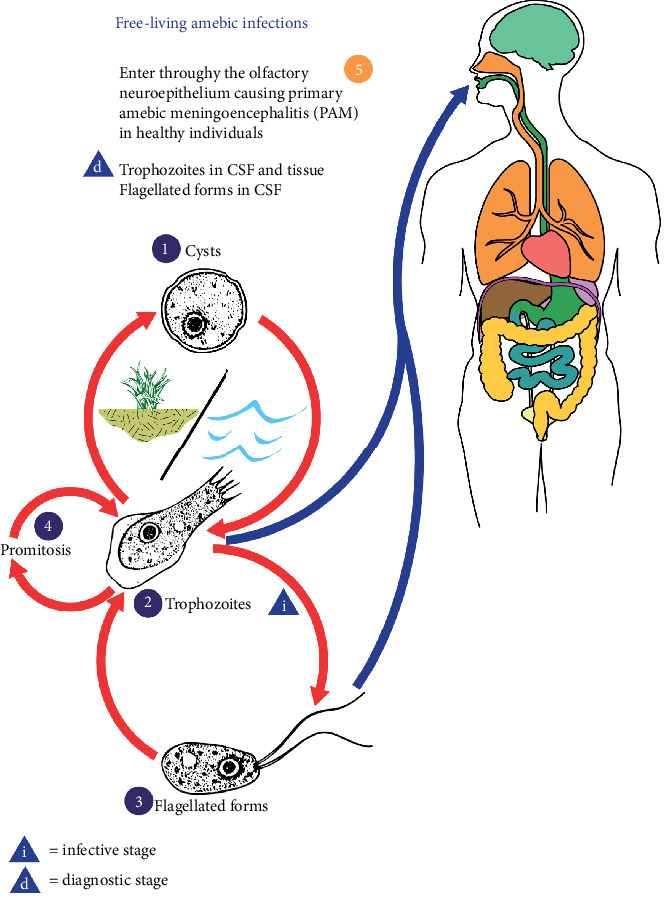
This illustration depicts the life cycle of *Naegleria fowleri*, the parasitic agents responsible for causing free-living amebic infections. Content Providers are CDC/ Alexander J. da Silva, PhD, and Melanie Moser. *N. Fowleri* has 3 stages in its life cycle: (1) cyst, (2) trophozoite, and flagellate (3). The only infective stage is the trophozoite which is 10-35 *μ*m long with a granular appearance and a single nucleus. They replicate by binary division during which the nuclear membrane remains intact (promitosis) (4). Trophozoites infect humans or animals by penetrating the nasal tissue (5).

The genus Naegleria is composed of over 30 different species made of a group of free-living ameboflagellate found in diverse habitat [1]. But only Naegleria fowleri has been isolated from the humans and a known agent for primary amoebic encephalitis. N. fowleri is a thermophilic amphizoid amoeba, single-celled microbe, commonly found around the world in warm freshwater [1]. Environmental conditions, including desiccation, salinity, and chlorination, affect their survival [2]. It can exist in a free-living state in water or soil or as a pathogen in human hosts, hence the term amphizoid. Naegleria spp. have been isolated from freshwater lakes, ponds, domestic water supplies, swimming pools, thermal pools, soil, and dust. Human disease caused by N. fowleri was first reported by Fowler and Carter in South Australia [3]. Although most commonly seen in southern states in the United States, it is essential to keep this clinical suspicion in mind regardless of geography, as presenting symptoms can be very similar to classic bacterial meningitis. Symptoms include fever, headaches, nausea along with nuchal rigidity, and sometimes seizures. As per the CDC, an infection can occur by recreational water activities, but not by drinking contaminated water with Naegleria. However, there have been reports of disease via nasal lavage. The majority of infections in the United States are prevalent in southern states arising in summer months, where the organism is found in geothermal water such as hot springs, lakes, poorly maintained swimming pools, and recreational waters. N. fowleri has the best growth at higher temperatures up to 115°F (46°C). The three morphological stages in the life cycle include a trophozoite, flagellate, and a cyst [4] (Figure 1). The trophozoite is the feeding and dividing stage for humans; however, the cyst can also enter through the nares and convert to trophozoites [4]. They attach to the nasal mucosa and then migrate through olfactory nerves across the cribriform plate and penetrate the brain tissue.

Through cytotoxic effects, the organism starts tissue destruction of nerve cells and ingestion of erythrocytes initiating an inflammatory response that consists of neutrophils, eosinophils, and macrophages. The pathologic mechanism includes proteinosis and possible lysis that contributes to neuronal demyelination, and destruction and lysis of erythrocytes and surrounding nerve cells [5]. As a result, neuroimaging in PAM usually shows a single focus on infection in the frontal lobe along with diffuse cerebral edema and basilar meningeal enhancement [6]. The detection method includes direct visualization under a microscope of fresh CSF samples using Giemsa Wright or modified trichrome stain, antigen detection, PCR, or amoeba culture [7, 8]. As per the CDC, in the last ten years, out of 34 cases reported in the United States, 30 people were infected by recreational waters, and half of the examples in the U.S have occurred in Texas and Florida. Symptoms start with a median of 5 days after exposure to contaminated water. Due to the rarity of the infection and its close presentation to bacterial meningitis, 75% of diagnosis is made after the death of the patients [9].

## 2. Case Report

**Figure 2:**
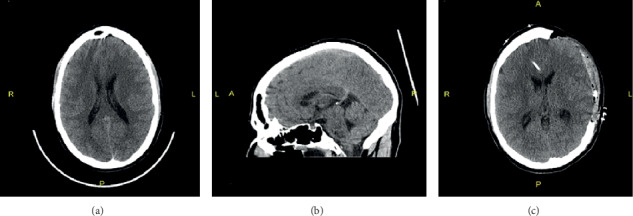
CAT scan on admission showing (a) axial, (b) sagittal, (c) post hemicraniectomy within 12 hrs. of admission. Note, there is diffuse sulcal effacement in (a), possibly reflecting hypoxic-ischemic encephalopathy Vs meningitis.

A 29-year-old Caucasian male with a past medical history of Epstein-Barr virus infection in childhood presented to AtlantiCare Regional Medical Center in New Jersey, Atlantic City division, with a sudden altered level of consciousness and fevers up to 103°F. Upon initial assessment, the patient was somnolent, altered, and not able to follow any commands. The mother provided the history given the clinical condition of the patient. She stated that the patient just returned five days ago from Texas, where he went on vacation. Also pertinent was that he worked outdoors for the New Jersey Department of Environmental Protection in water testing facility. Five days before his presentation, the patient was exposed to recreational waters at a surfing park in Waco, Texas. On physical examination, the patient was febrile and comatose with a Glasgow Coma Scale of 7. His blood pressure was 153/75 mmHg with a pulse rate of 77 beats per minute and an oral temperature of 103°F. Upon examination of lungs, abdomen, and extremities, no abnormality was noted. No features of chronic illnesses or palpable lymph nodes were appreciated. On his neurologic exam, he was altered, moving all extremities with a positive Brudzinski sign. We sedated and intubated the patient for airway protection in the emergency department and admitted him to the neurocritical care unit. A computerized tomography (CT) scan of the brain showed no evidence of acute intracranial infarction or hemorrhage; however, there was sulcal effacement concerning for hypoxic/ischemic encephalopathy versus meningitis (Figure 2). Given the patient's presentation, the suspicion was high for bacterial meningitis versus viral encephalitis. Blood cultures, serologies for West Nile virus, Rocky Mountain spotted fever, cytomegalovirus, Herpes simplex virus, Lyme's disease, and an acute Epstein-Barr virus panel were all obtained. The patient received 10 milligrams of IV dexamethasone. Vancomycin and meropenem plus acyclovir were initiated for broad-spectrum coverage. In the intensive care unit, a lumbar puncture demonstrated an opening pressure of 46 mmHg, and the procedure was aborted. We placed a right frontal external ventricular drain (EVD) that revealed an opening pressure of 15 mmHg. During this time, we collected the CSF and sent it for further studies. Cerebrospinal fluid studies showed a white cell count of 1021 cells/uL, red blood cell count of 1830 cells/uL, the glucose of 98 mg/dL, and a protein of 174 mg/dL. His peripheral white blood cell count was 11400 cells/uL (83% segmented neutrophils). The EVD was left open to drain at a pop off setting of 0 mmHg. An MRI of the brain done post admission showed no evidence of acute infarction, and the ventricular volume was normal.

On day one post admission, the patient's ICP continued to remain elevated, sustaining at 30-40 mmHg despite EVD placement and multiple interventions, including hyperventilation, hyperosmolar therapy with 3% saline, and a paralytic agent. A phenobarbital-induced coma with burst suppression was started, which initially dropped the ICP to 10 mmHg, but the ICP soon rebounded and increased to 40 mmHg. A left decompressive hemicraniectomy was then performed, which decreased the ICP to 15-20 mmHg. The patient's initial laboratory workup was still pending, including blood, fungal cultures, CSF gram stain/cultures, and viral PCR. We sent out the tick-borne disease panel, and doxycycline, azithromycin, and atovaquone were empirically added to the aforementioned antibiotic therapy.

On day two post admission, the patient's left scalp was tense, and the right frontal EVD again demonstrated elevated ICP. The elevated ICP was refractory to continued maximal medical management, and the neurosurgery team performed a right decompressive hemicraniectomy. Patient cultures and the laboratory workup, as mentioned above, were all negative. Given patient's presentation, with clinical signs and travel history, amphotericin B and fluconazole were added to the treatment and more CSF sample was sent for parasite smear to test for possible amoebic meningoencephalitis at our institution.

**Figure 3:**
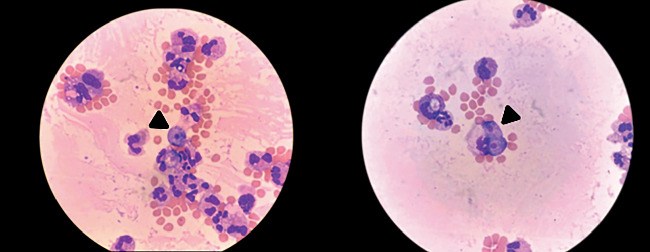
Naegleria trophozoites were rapidly identified by their large, round- to pear-shaped cells with a prominent, dark nucleus, and scattered vacuoles. Images of Naegleria fowleri on Giemsa Wright-stained CSF slides (1000x, oil immersion). Black arrowheads point to Naegleria trophozoites with a background of mostly RBC and some neutrophils.

**Figure 4:**
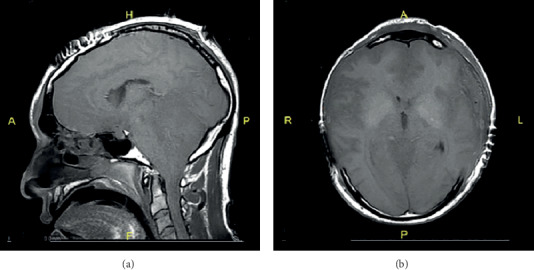
MRI post 72 hours of admission. After uncomplicated wide bilateral craniectomies. (a) The sagittal view showing transtentorial herniation with stretching and obliteration of the fourth ventricle. (b) Note on the axial view showing diffuse swelling with loss of gray white junctions and absence of ventricular space.

On the third day post admission, parasite smear results came back concerning for amoeba infection. We sent samples to the CDC, which confirmed the diagnosis of Naegleria fowleri (Figure 3). Vancomycin and acyclovir were discontinued, and we started the patient on amphotericin B, fluconazole, and azithromycin. Despite the bilateral craniectomies and other interventions, the patient's ICP remained elevated, and he started to demonstrate signs of brain death with MRI revealing transtentorial herniation without uncal or subfalcine herniation (Figure 4). The physical exam off sedation and nuclear medicine cerebral blood flow study both confirmed findings consistent with brain death, and we terminally extubated the patient.

## 3. Discussion

This case presents with a first-ever reported case of PAM in the state of New Jersey, and it highlights the importance of keeping a clinical suspicion in mind regardless of geographic location. The infection primarily affects younger people who are exposed to warm recreational freshwater in southern states during the summer months [10]. Increased incidence in young adults and children is likely more related to water-related activities as they are more likely to dive, submerge the head in water, or engage in water sports like water surfing, water rafting, and skiing that can force water up to the cribriform plate from the nasal cavity. The patient presented with signs including fevers, chills, photophobia, confusion, with a tripod sign that indicates respiratory distress and meningeal irritation, which required him to undergo intubation immediately as his GCS was 7.

The diagnosis of PAM is challenging because of the similarities between the presenting symptoms of PAM and other conditions, including bacterial meningitis. PAM is rapidly progressive and has a devastating disease course that usually occurs within 6-17 days after initial exposure. This can lead to death if not promptly diagnosed [11]. Specific diagnostic tools and general increased awareness of PAM are required to initiate treatment sooner. In the few cases of PAM that were successfully managed in the past, a correct and prompt diagnosis was made [12, 13]. History of water exposure is critical in diagnosing PAM. The current case presented with clinical signs and symptoms similar to previously documented cases [14, 15]. Symptoms of PAM are nonspecific symptoms and include severe headache, high-grade fever, confusion with altered level of consciousness, and neck stiffness. Death, in most cases, is due to increased intracranial pressure and herniation, as seen in our patient (Figure 4).

N. fowleri induces pore-forming proteins that cause lysis of mammalian cells on contact and secrete cytolytic molecules, including acid hydrolases, phospholipases, neuraminidases, and phospholipolytic enzymes that degrade mammalian tissue [1]. The organism also synthesizes regulatory surface proteins that confer protection to amoeba against complement-mediated lysis and other cytotoxic substances, thus evading the host immune system [1]. Laboratory findings in patients with PAM are characterized by increased leukocytes, which are predominantly polymorphonuclear cells. CSF may also be purulent with marked leukocytosis, increased protein, and reduced glucose level [14, 15]. In most cases, neuroimaging tests in the early stages of the disease did not reveal any brain abnormalities [16]. However, strong clinical suspicion of PAM with a previous history of water exposure should guide the management of the patient's presentation in the absence of bacteria or fungi and CSF specimens. Measures to avoid this fatal infection include avoiding water-related activities in freshwater lakes, rivers, or natural hot springs with high water temperatures.

Its low incidence limits effective treatments for an N. fowleri infection, and current treatment strategies are based on case reports and in vitro animal studies. Amphotericin B is a pharmacologic treatment with potential efficacy when started early. It acts by binding to ergosterol in the cell membrane with pore formation that leads to cellular lysis. In vitro studies show a concentration of at least 0.1 *μ*g/mL is needed to suppress >90% of amoeba growth, and 0.8 mg/mL is required to achieve 100% amoebicidal capability [17, 18]. Based on this, intravenous doses of amphotericin B of 0.25 mg to1.5 mg/kg/day are recommended in adults, while doses ranging from 0.5 to 0.7 mg/kg/day is recommended in pediatric patients [18]. CDC currently recommends intravenous or intrathecal (if ICP is increased) amphotericin B at doses of 1.5 mg/kg/day in two divided doses for three days, followed by 1 mg/kg/day once daily for an additional eleven days with a total of 14 days of therapy [18]. Fluconazole, an antifungal agent, may be effective at a dose of 10 mg/kg/day daily for 28 days. Fluconazole then synergistically works with amphotericin B by inhibiting ergosterol synthesis. Also, azithromycin, the macrolide antibiotic, has in vitro activity against N. fowleri and is recommended to be used at 10 mg/kg/day once daily (maximum 500 mg daily dose) for 28 days [9]. Miltefosine, a drug effective against leishmaniasis, is another potential treatment for PAM recommended by the CDC at doses of 50 mg orally two to three times daily (based on body weight) with a maximum dose of 1.5 mg/kg/day for a total of 28 days [18]. The exact mechanism is not precise; however, it is believed to interact with phospholipids and steroid molecules in the cellular membrane.

PAM is almost always uniformly fatal. Early diagnosis and aggressive treatment are crucial for recovery. In addition to pharmacologic management against amoeba, control of high intracranial pressure, systemic inflammation, and possible induction of moderate hypothermia are a multidisciplinary approach in treating PAM [19]. Neuroprotective management principles following any cerebral insult are based on maintaining adequate cerebral perfusion, tempering oxygen consumption, and lowering ICP with mild to moderate hypothermia (32-34°C). These steps reduce reactive oxygen species formation and proinflammatory cytokines halting neuronal apoptosis [20]. Of the 143 reported PAM cases in the United States from 1962 to 2017, this is the first case reported in New Jersey. A high clinical suspicion is required to diagnose PAM for patients presenting with nonspecific symptoms similar to bacterial meningitis. History and physical exams are crucial in the diagnosis of PAM as early as possible so that appropriate treatment can be initiated, along with aggressive neurological critical care management.
